# Fused-Deposition-Material 3D-Printing Procedure and Algorithm Avoiding Use of Any Supports †

**DOI:** 10.3390/s20020470

**Published:** 2020-01-14

**Authors:** Gianluca Barile, Alfiero Leoni, Mirco Muttillo, Romina Paolucci, Gianfranco Fazzini, Leonardo Pantoli

**Affiliations:** 1 Department of Industrial and Information Engineering and Economics (DIIIE), University of L’Aquila, Piazzale Pontieri 1, Monteluco di Roio, 67100 L’Aquila, Italy; gianluca.barile@graduate.univaq.it (G.B.); alfiero.leoni@graduate.univaq.it (A.L.); mirco.muttillo@graduate.univaq.it (M.M.); romina.paolucci@graduate.univaq.it (R.P.); 2 Research & Development department, 3DPRN, 65013 Pescara, Italy; info@3dprn.com

**Keywords:** additive manufacturing, 3D printing, supports, FDM

## Abstract

The three-dimensional printing of complex shapes without using supporting structures is the most attractive factor of merit in current additive manufacturing because it allows to drastically reduce printing time, and ideally nullify postprocessing and waste material. In this work, we present an innovative procedure and algorithm (Print on Air, PoA) for additive manufacturing that, relying on sensing systems embedded into the three-dimensional (3D) printer (e.g., temperature and speed sensors), aims at generating a printing sequence capable of a self-sustaining bridge and overhang structures. This feature was achieved by splitting the actual floating area of the layer where the aforementioned structures are in many subsections. Each is generated with a negligible floating surface and printed in a well-determined sequence with accurate temperature and speed profiles. Therefore, each subsection is formed without the need for scaffolding, simultaneously acting as a supporting structure for the following subsection. The array of subsections constitutes the actual bridge or overhang structure. The proposed method can be used for any object, including very long bridges or convex surfaces. The revolutionary method is here reported and evaluated in order to show its applicability in any condition. Although the study was conducted in a Fused Deposition Material (FDM) environment, it can certainly be adapted to other manufacturing environments with adequate modifications.

## 1. Introduction

Additive manufacturing is becoming the primary solution for prototyping and realizing three-dimensional (3D) objects. Nowadays, additive techniques have the potential to replace classical manufacturing technologies thanks to the use of innovative materials and design capabilities. Traditionally, three-dimensional objects and parts were obtained by “subtracting” material from a larger block through digging and cutting, inheriting all mechanical limitations that arise, for instance, when closed and empty objects have to be realized. Indeed, this kind of shape is usually manufactured with molds. The basic idea of additive techniques is the creation of an object layer by layer, deposing and superimposing materials only where materials are actually needed. Many depositions methods (also known as 3D-printing techniques) have been defined.

Summarizing the main benefits and advantages relative to additive manufacturing, the following points can be remarked:

- Very low manufacturing costs for low-volume productions (no mold production needed);
- lower production time for low-volume productions (no mold iterations needed);
- waste reduction for better sustainability;
- avoiding the use of complex equipment or production lines for object realization;
- no more need for warehouses since “zero miles” products can be realized; and
- capability of realizing the same object with several materials, hence different mechanical properties.

In addition, this new concept allows to overcome traditional mechanical limitations in assembling objects.

Three-dimensional-printing technologies are nowadays a concrete alternative for production. At the research level, great efforts are spent [1,2,3,4,5,6,7] with the aim of further improving printing capabilities and reliability with novel materials [8,9], novel electronic components and sensors [10,11,12,13,14], and product quality. Different types of additive-printing technologies have been defined in the literature, and each of them experienced some drawbacks, mainly due to technology limitations.

Among them, a primary role is reserved to the Fused Deposition (FDM) technique that creates an object from the bottom to the top by heating a thermosensitive filament to a temperature close to its melting point and extruding it layer by layer. Considering the high number of printable materials and structures that can be realized, together with the peculiarities of FDM technology, it is possible to achieve various and interesting physical proprieties, such as flexibility, toughness, thermal resistance, and electrical conductivity.

A generic and standard FDM printing process can be summarized in the following steps:

- Slicing process. This is a preliminary yet fundamental setup process in which the object to be printed is analyzed by dedicated software (slicing software) that determines the deposition layers and their orientation. In addition, the same software calculates extrusion paths and places supporting structures where needed according to user-defined constraints.
- Printing process. The precalculated tool path is filled, layer by layer, with strands of thermosensitive material. For the sake of clarity, we define “layer” as the ensemble of building material (be it the actual object or a supporting structure added by the slicer) that is extruded at the same height; each layer is characterized by predetermined (during the slicing procedure) constant thickness. In this same phase, supports are printed together with the object: they can be made out of the same material as the printed object or different soluble materials. In the former case, weak spots are added to the supporting structure in order to promote their removability in the following stages by decreasing their adhesion to the actual object of interest (at the expense of geometrical accuracy).
- Support removal. The support material added to mitigate shape flaws has to be removed according to the adopted printing solution. A mechanical removal process is necessary if the supporting material is the same as that of the object, while special detergents can be used to dissolve them if soluble supports are realized.
- Finishing process. Usually, this is unnecessary if supports have not been used, since the final overall quality is valid if a good printing resolution has been used. Otherwise, if supports have been printed and removed, a cleaning and sanding process is often necessary to improve the object finish and bring its dimensions within specs.

**Figure 1:**
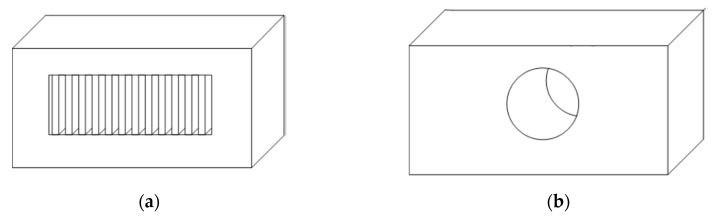
(**a**) Support-based object and (**b**) supportfree object.

According to the above, the capability of printing without supports (refer, for instance, to Figure 1) is therefore a key feature to improve the overall quality of the product and save material and time. In some cases (e.g., closed surfaces), internal supports cannot be removed from the final product since they become inaccessible once the printing process is completed.

These motivations have inspired this work that focuses on the definition of a new printing procedure and algorithm that allow avoiding the use of any supporting structure regardless of object shape. In this solution, each hanging section of a layer is split into many subsections. Each is then deposited following a specific sequence so that it simultaneously produces part of the final object and support for the following subsection. A key point of the proposed approach is that the slicing procedure that handles bridges and overhangs was implemented according to a nonstandard custom method that is shown in the following sections.

## 2. Support-Based 3D Printing

**Figure 2:**
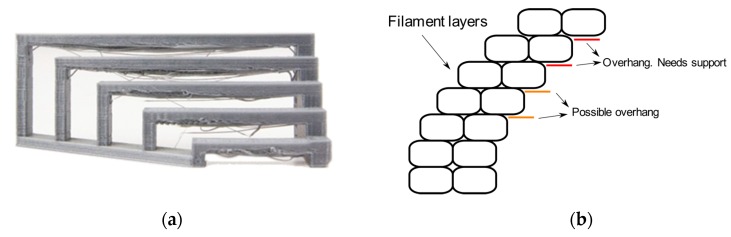
Limitation examples: (**a**) bridging; (**b**) overhangs.

**Figure 3:**
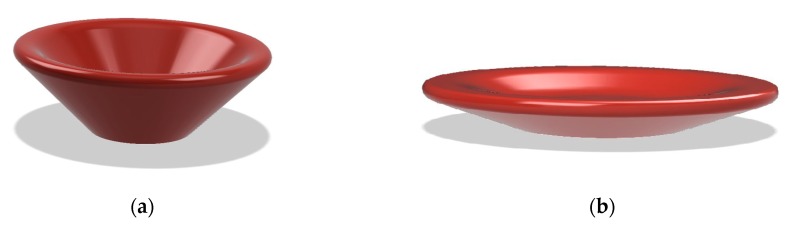
Object with overhang (**a**) less than 50 degrees, where generally no support is needed, and (**b**) greater than 50°, where supports are needed.

In the standard FDM 3D printing process, the 3D object is built by means of layering melted plastic threads that are extruded following a predetermined path. Each layer is deposited with an offset (which can be constant as well as variable) from the previous. Since gravity inevitably acts on the hot extruded plastic, each deposited strand, in order to maintain the supposed shape, needs to be able to solidify without drooping or deforming. This is why one of the main limitations of the FDM 3D printing process (but also of other processes like stereolithography (SLA)) can be identified in the capability of printing all these structures where a section of the n-th layer is not supported by the (n − 1)-th layer. These shapes are called overhangs and bridges (Figure 2a,b, respectively). Overhangs are typically negligible if the angle formed with the printing Z axis is less than 50° (see Figure 3). For any steeper angle, geometrical inaccuracy occurs if proper precautions are not taken.

**Figure 4:**
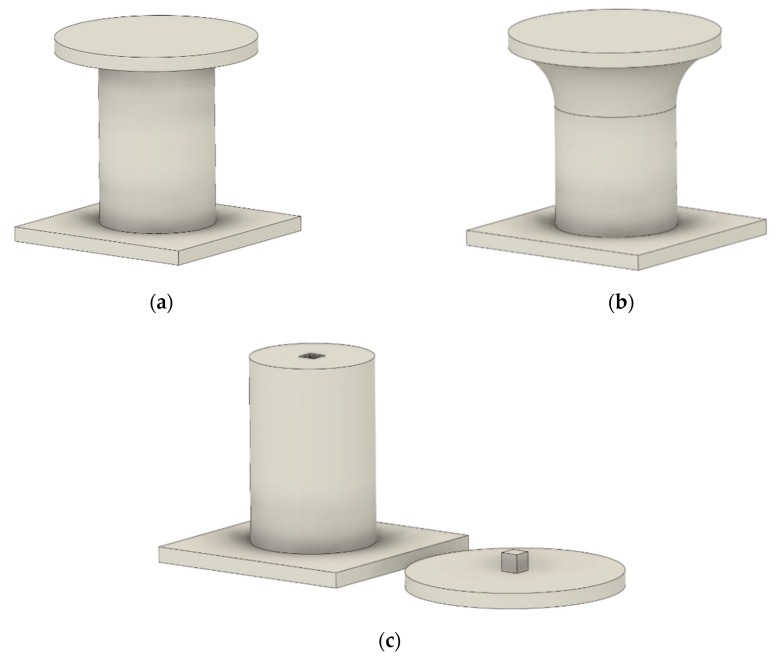
(**a**) Object with poor printability; (**b**) printable object filleting the floating area; (**c**) printable object splitting critical area from main body.

There are a number of possible approaches to deal with this limitation. The first and most straightforward is “design for printability” [15,16,17]. This technique has to be employed from the object-design stage and consists of using a number of design criteria with the aim of simultaneously generating a functional and supportless 3D printable object (for instance Figure 4a,b). This can be a complicated process and often impossible to achieve.

A similar approach is to split a given design into many sub-blocks, each easily 3D-printable (Figure 4c). Indeed, the authors in [6] proposed a sophisticated decomposition algorithm for the generic 3D model with overhangs in order to split each critical part and separately print it. With this method, no support has to be generated, resulting in saving material, but it requires a postprocessing step for part assembling, sticking, and polishing, which is a time-consuming operation that could affect the quality of the printed object. Wu C. et al. [18] presented an enhancement of the model-partitioning technique proposed in [6] by combining the latter with a custom 6-DOF robotic printing arm. There, the object was entirely printed without splitting apart, and the 3D printer bed was oriented according to the partitioning algorithm that evaluated the best printing orientation for each highlighted critical part in order to avoid overhang issues. The major drawback of such a technique is the need for a special 3D robotic arm with complex cinematics since the method could not be adapted on a regular 3D printer. Moreover, this technique is not always applicable because of extruder encumbrance, which in some cases limits robotic-arm movements between the preprinted parts of the model.

Another viable solution is to allow software to generate supporting traces to hold floating structures. There are two types of supports: the nondissolvable, or standard, and the dissolvable [19,20,21]. The former category allows a single extruder setup to generate both supporting structures and the actual object of interest.

Although it allows to use very simple machinery, generating supporting structures on the basis of the same material as the main object implies complicated manual postprocessing that is both time-consuming and unable to leave clean surfaces. This leads to poor geometrical accuracy. On the other hand, using soluble scaffolding simplifies actual postprocessing (the object only needs to be submerged in water or acetone) and allows to obtain very smooth and accurate surfaces once removed. The downside is the cost of the material (typically much higher than the cost of the plastic which is used to build the actual object) and, most importantly, the necessity to have at least a dual-extrusion setup (higher machinery costs). Both aforementioned methods have two more important limitations, which are the increased printing time and increased material waste.

## 3. Proposed Algorithm and Printing Method

To overcome the above problems, here we propose a very simple approach that allows supportfree 3D printing with any kind of 3D printer. The approach was applied to the slicing process, allowing to print both supportless bridges and very steep overhangs.

**Figure 5:**
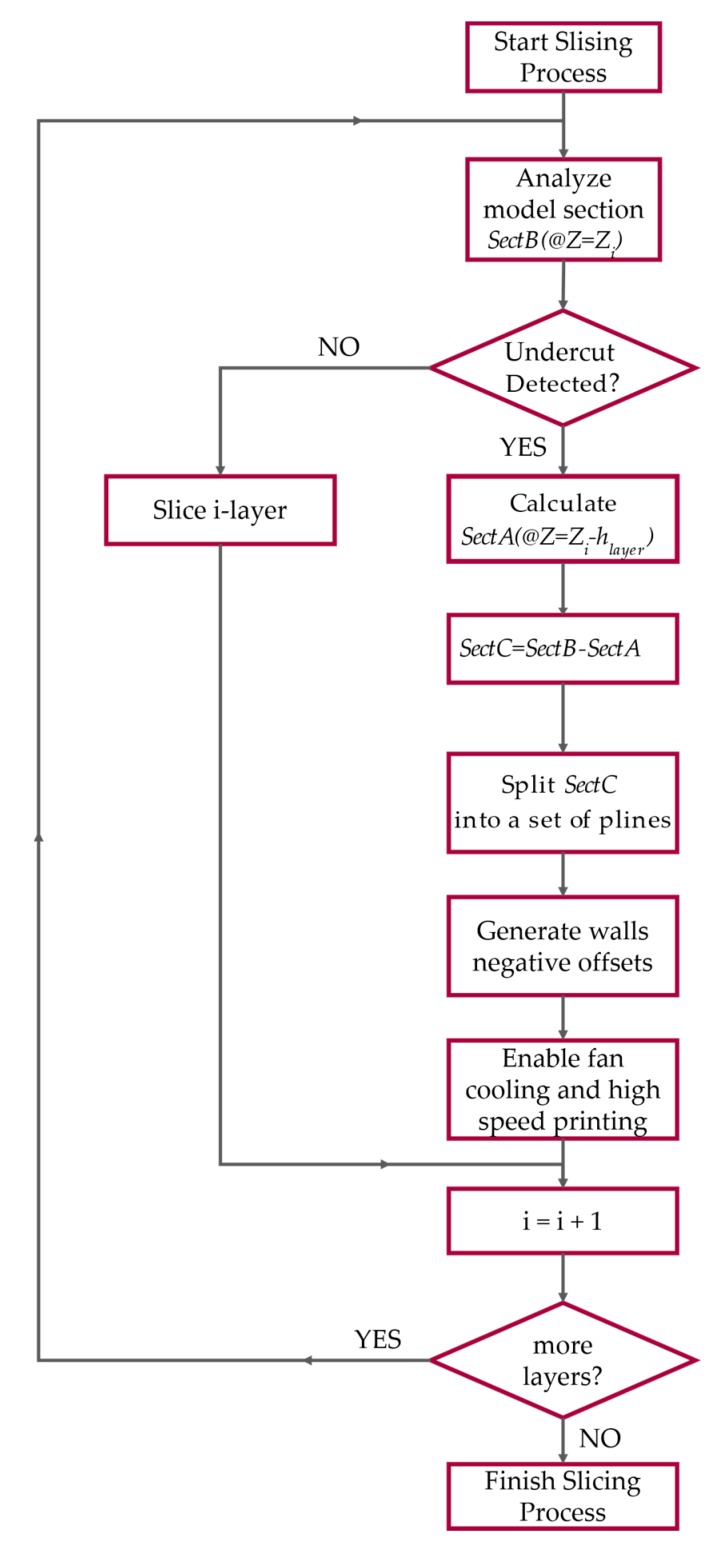
Flowchart of proposed algorithm.

**Figure 6:**
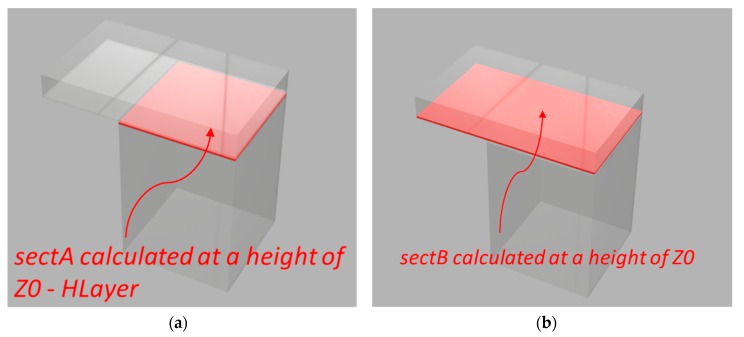

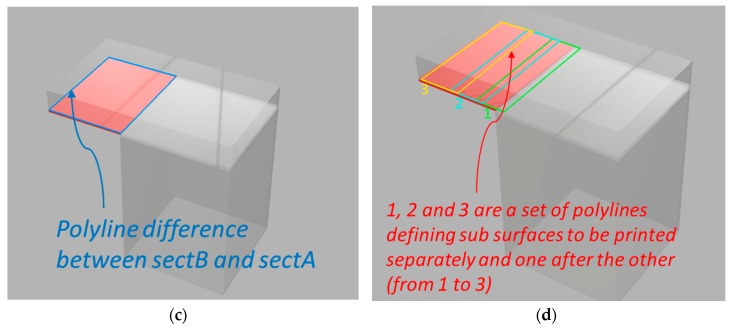
Proposed printing approach divided into four steps (**a**–**d**).

To better understand we outline the working principle of the proposed method, the slicing algorithm flow of operation. The flowchart of the method is given in Figure 5. When, during any slicing procedure, an undercut is detected at height $Z_{i}$, the following algorithm takes place, substituting the standard one:

1. Calculate the surface of the plane that aws printed just before the undercut at height $Z_{i-1}=Z_{i}-h_{layer}$, where $h_{layer}$ is the deposition thickness of each layer (or at least the deposition thickness of the previous layer). This surface is named sectA (Figure 6a).
2. Calculate model surface at height $Z_{0}$. This section is called sectB (Figure 6b).
3. Compute difference sectC=sectB−sectA. The result is a polyline that represents the undercut boundary (Figure 6c).
4. Generate a set of polylines that cover all overhang area sectB−sectA, and obtain the list of polylines (listOffsetSezA). The offsets from sectA for each polyline are generated for steps smaller than the extruded filament width until it reaches the external boundary of *sectC*. The computed offsets are such that each extruded polyline inevitably superimposes the previous one for a very small fraction of a millimeter (Figure 6d). In other words, each polyline is both part of the model and acts as a supporting structure for the next one.
5. A negative offset is calculated for the external perimeters (external walls) that are printed at the end. The offset amount is based on the actual physical properties of the machinery that is used.
6. For each obtained path, the polyline is printed at a sustainable extruder head speed with active fan cooling, so that the deposited thermoplastic material can sufficiently harden to sustain the next offset polyline, until the end of the overhang part is completely printed.

**Figure 7:**
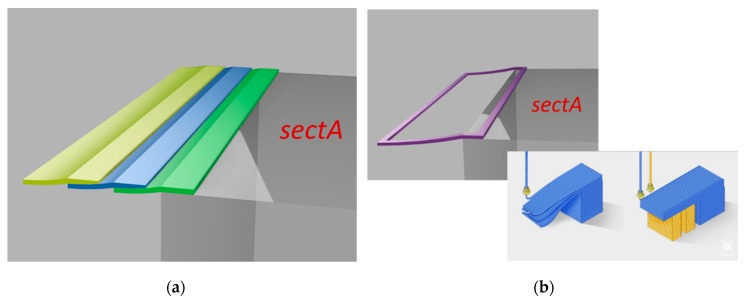
Comparison between proposed algorithm and typical slicing result. (**a**) Overlapping procedure of proposed method; (**b**) supportfree result without proposed method and nonfree-support results.

Once the polylines defining each subsurface are determined, they are filled in a sequence so that each one acts as a support for the next one. Only at the end of this procedure are external perimeters added. A typical slicing procedure would instead start from perimeters of the entire object needing some supporting structure (Figure 7).

During the deposition phase along a generic direction, there is the need for punctual active material cooling with fans in order to facilitate material hardening without collapse. Another requirement for the algorithm to work properly is a per-material-basis cooling profile. This means that, according to the printing material, an adequate cooling profile has to be studied. This is not a limiting factor since, typically, such a profile is already given by the vendor themselves.

## 4. Experiment Results

**Figure 8:**
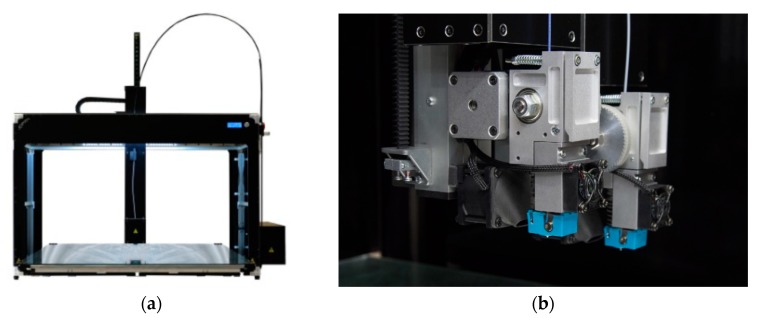
(**a**) Testing 3DPRN LAB H5 FDM printer; (**b**) dual-extrusion setup utilized for test.

The presented solution, whose preliminary result was presented in [22], was implemented into the Tips slicing software (3DPRNWARE), which is a modified version of open-source slicing engine Slic3R, and compared to the typical supportless slicing procedure. The used printer in our tests is a 3DPRN H5 [23] with a dual tilting extruder setup, reported in Figure 8, enabling to compare standard techniques with the proposed one using the same platform. Cooling and speed were properly tuned for the material.

**Figure 9:**
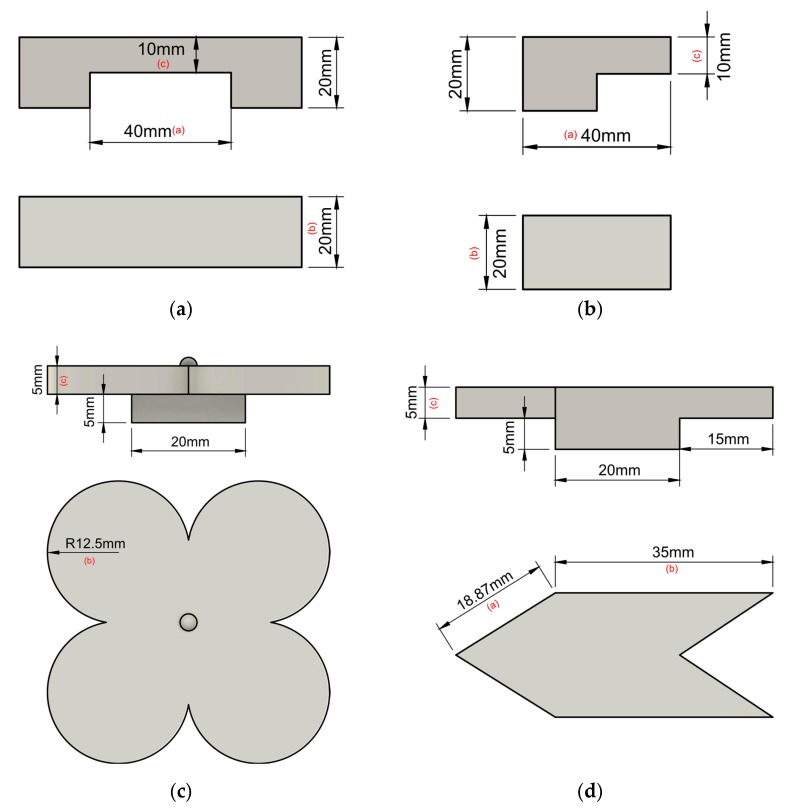
Test samples printed for analysis: (**a**) bridge, (**b**) rectangular 90° overhang, (**c**) circular 90° overhang, (**d**) triangular 90° overhang (pictures not in scale with each other. Letters besides dimensions refer to measured dimensions reported in Table 1).

For analysis, four different specimens were considered, each with a different form factor, to validate the proposed algorithm in different scenarios. Three of them were a 90° overhang (with a circular, triangular, and rectangular floating surface; see Figure 9b–d), while the last one was a bridge (see Figure 9a). CAD (computer aided design) dimensions of the samples are reported in Figure 9.

**Figure 10:**
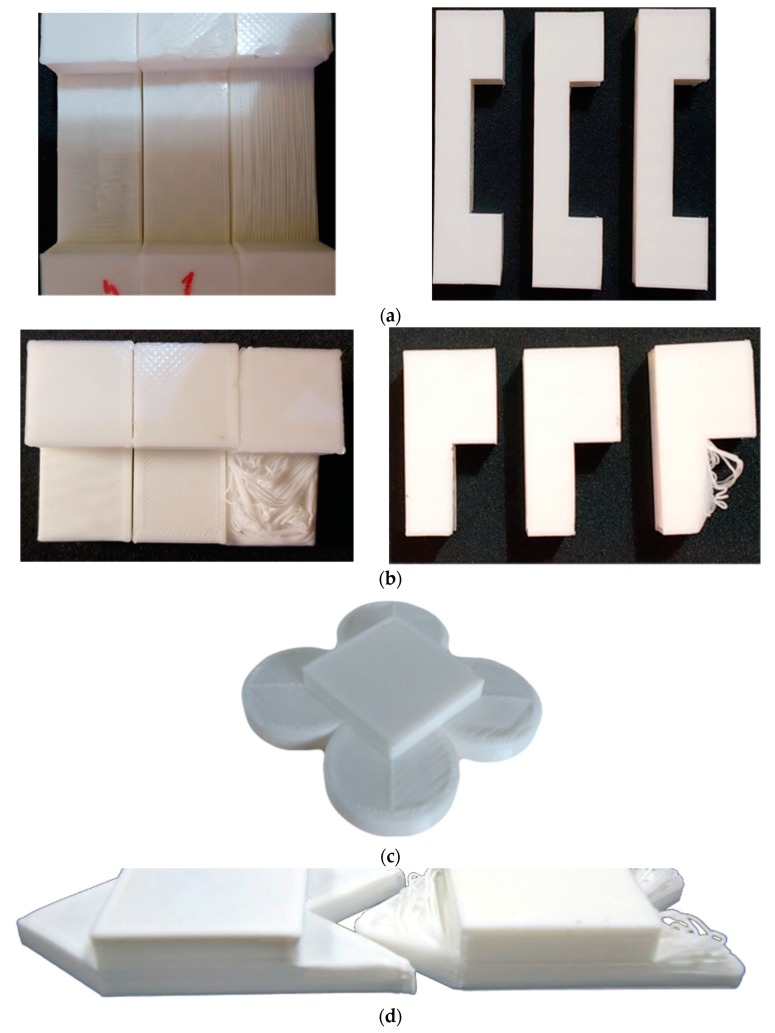
Printed samples: (**a**) bridge structure (left to right): Print on Air (PoA), supports, supportless; (**b**) rectangular overhang (left to right): PoA, supports, supportless; (**c**) circular overhang printed with PoA; (**c**) triangular overhang (left to right): PoA, supportless.

All aforementioned models were printed 10 times with supports (on the basis of the same material as the main object), without supports, and with the Print on Air algorithm. The layer height was set at a constant value of 0.2 mm, while speed, acceleration, jerk, and cooling were automatically set by the slicer. A highlight of the printed models is given in Figure 10, where the effectiveness of our algorithm is visually signified.

Test samples were measured with a digital micrometer (±3 µm accuracy). For each sample, measurements were taken at different points of the surfaces, and the mean value was extracted. The mean value across the 10 samples was evaluated and reported in Table 1. Measured dimensions a–c are highlighted in the CAD models of Figure 9.

For the sake of completeness, the lateral dimensions (dimensions a and b) were also added to the table; however, unsurprisingly, they did not differ from each other or from the ideal one across all conditions. Interesting results came from the thickness of the floating surfaces. Starting from the overhangs, no effective measurement was possible for the unsupported samples. Indeed, as also visible from Figure 10, floating layers were completely drooped, and the piece would be unusable in almost all working applications. PoA, on the other hand, was capable of remaining within one-layer error (0.2 mm) from the ideal dimension. Supposedly, this error came from the cooling deformation of the plastic (wavy-surface finish) rather than actual material droop; hence, a better cooling profile could further improve the results. Moreover, for larger pieces, this issue is inherently reduced since the extruder physically moves farther away from each deposited strand, reducing unwanted heat exchanges between itself and printed sections. Thickness accuracy of the supported samples was the best, regardless of conditions, but we added a solid sacrificial interface for these prints between printed object and the actual scaffold. Therefore, what we reported here is the best-case scenario (even soluble supports would not improve dimensional accuracy). It is important to put in evidence the extra amount of printing time and wasted material that this method requires compared to PoA.

Taking into consideration the bridge structure, even unsupported standard slicing procedures are capable of effectively handling this situation, given that the filament strand was fixed at both ends before the extruder changed direction. This reveals that the proposed procedure is best suited for overhangs rather than bridges.

## 5. Conclusions

In this work, we presented a novel method for FDM 3D printing that enables to print floating structures without supports. The method is relatively simple to handle, and only uses sensing devices and structures already embedded into a standard machine without the need of extra hardware. The proposed approach can be applied to any object, including long bridges and convex surfaces. The algorithm was accurately tested both with differently shaped overhangs and bridges. From analysis, we can conclude that, regardless of shape, the supported structures showed the best accuracy across almost all measurements. However, given that, from a geometrical point of view, the accuracy of the obtained shapes with the proposed algorithm was fully comparable with the previous, considering the saved material, time, and postprocessing, our proposal is a valuable tool.

## Figures and Tables

**Table 1:** Measurement results for the four different printed specimens.

	Rectangular 90° Overhang			Circular 90° Overhang			Triangular 90° Overhang			Bridge		
	*PoA*	*Supports*	*No Supports*	*PoA*	*Supports*	*No Supports*	*PoA*	*Supports*	*No Supports*	*PoA*	*Supports*	*No Supports*
*Dimension a (mm)*	40.10	40.12	40.16	-	-	-	18.78	18.81	18.72	40.20	40.11	40.07
*Dimension b (mm)*	20.02	20.06	20.26	12.55	12.61	12.53	34.89	35.06	34.81	20.03	19.99	20.03
*Dimension c (mm)*	10.21	10.03	N.A.	5.15	5.06	N.A.	5.26	5.03	N.A.	10.22	10.09	10.33
*Time (hh:mm)*	00:28	00:38	00:28	00:41	01:04	00:40	00:16	00:25	00:16	00:56	01:12	00:55
*Useful plastic (g)*	≈6			≈7			≈3			≈10		
*Wasted plastic (g)*	0	≈2	0	0	≈3	0	0	≈1	0	0	≈5	0
*Post processing*	No	Yes, hard	No	No	Yes, hard	No	No	Yes, hard	No	No	Yes, hard	No
